# Study on Fabrication and Properties of Polyvinyl Alcohol/Chitosan Nanofibers Created from Aqueous Solution with Acetic Acid and Ethanol by the Electrospinning Method

**DOI:** 10.3390/polym16233393

**Published:** 2024-11-30

**Authors:** Thi Hong Nhung Vu, Svetlana N. Morozkina, Roman O. Olekhnovich, Aleksandr V. Podshivalov, Mayya V. Uspenskaya

**Affiliations:** 1 Faculty of Basic Sciences, Vietnam National University of Forestry at Dong Nai, Trang Bom 76000, Dong Nai Province, Vietnam; 2 Kabardino-Balkarian State University, Chernyshevskogo 173, 360004 Nalchik, Russia; i_norik@mail.ru; 3 Center for Chemical Engineering, ITMO University, Kronverkskiy Prospekt, 49A, 197101 St. Petersburg, Russia; podshivalov2005@mail.ru; 4 Civil Engineering Institute, Peter the Great St. Petersburg Polytechnic University, Polytechnicheskaya, 29 B, 195251 St. Petersburg, Russia; r.o.olekhnovich@mail.ru (R.O.O.); mv_uspenskaya@mail.ru (M.V.U.); 5 Institute of Chemistry, St. Petersburg State University, 7-9 Universitetskaya Embankment, 199034 St. Petersburg, Russia

**Keywords:** poly(vinyl alcohol), chitosan, biomaterials, electrospinning, fiber technology, targeted drug delivery system

## Abstract

The development of nanofibers with incorporated biologically active molecules with a targeted mode of action is a current research trend. Potential materials for the development of such systems include poly(vinyl alcohol) (PVA) and chitosan (CS) nanofibers, which are traditionally fabricated by the electrospinning of aqueous solutions of these polymers with acetic acid. To improve drug integration, ethanol was added to the binary-solvent system. This results in several important data: noticeable shifts in the solvent system’s solubility parameter, the interaction of the various component forces, and optical and rheological properties of the PVA-CS solution. The use of ethanol in the electrospun solution also contributes to adjusting the solubility parameters of the solution in the Teas graph, maintaining the “*f_h_ − f_d_*” in the optimal region for the fabrication of PVA-CS nanofibers. Increasing the efficiency of PVA-CS nanofiber fabrication by electrospinning is quite difficult due to the requirements of solution parameters, technological parameters, and environmental parameters; however, this efficiency was increased in this work by 2 to 3 times with a more optimal PVA-CS nanofiber morphology. These results demonstrate that aqueous solution containing 4% PVA, 3% CS, 15% ethanol, and 45% acetic acid is optimal for increasing the nanofiber fabrication productivity, improving the morphology and diameter of PVA-CS nanofibers without changing in chemical bonds. The XRD spectrum revealed that the alterations in the crystal lattice and diameter of the PVA-CS nanofibers led to the variation in their thermal and tensile properties.

## 1. Introduction

The development of targeted drug delivery systems is an actual research direction in medicine. The nanofiber-based drug delivery system represents a system that works via the directed delivering of the drug to the diseased cell. This helps to reduce the dose of drugs and to avoid the wasting of drugs, thereby reducing the amount of the drug to be produced, and thus reducing the economic burden. In addition, this also allows us to minimize the side effects of the drug and/or the tendency to become drug-resistant in patients. PVA-CS nanofibers represent one of the potential targeted drug delivery systems.

Fabrication of PVA-CS nanofibers by electrospinning is a very attractive research direction in the field of drug integration for drug delivery purposes. PVA and CS polymers have been confirmed to be non-toxic, biosafe, biocompatible [1,2,3,4,5,6,7,8,9,10,11], and have a wide range of applications in the industrial, food, and especially medical industries [1,6,7,8,9,10,11,12,13,14,15,16]. The combination of PVA and CS improves the moisture-sensitive properties of PVA and the insolubility of CS, also increasing the stability of the system. In the drug delivery system, due to its hydrophilic nature, PVA helps the composite material easily pass through the body to the organs. Apart from that, due to its polysaccharide structure, being a basic and positively charged polyamine, CS is soluble only in the acidic environment of diseased cells in the body, and CS serves as a guiding system for drug delivery and release at the targets. Basically, healthy cells have a pH between 7.1 and 7.4 [14,15], whereas most diseased cell types have an acidic environment, and depending on the cell type and the condition of the injury, their pH can drop even to 4.7 in fracture-related hematomas [17,18,19,20]. Derived from chitin, CS is a linear polymer. Thanks to its advantageous characteristics, which include biocompatibility, biodegradability, non-toxicity, and antibacterial activity, it is one of the most widely used natural polysaccharides with a wide range of the uses in the field of biomedicine [21,22,23,24,25,26,27]. Additionally, this polysaccharide has been utilized as a base material for the synthesis of nanomaterials for drug delivery, including nanoparticles and nanofibers [10,11,21]. The solubility of CS in water and organic solvents severely restricts its range of applications and relevant fields. However, the active functional groups in CS are susceptible to chemical interactions [21,24,26]. Many polymers, including PVA, have been reported to be biocompatible with CS [9,10,11,24,25,28,29]. Thus, the combination of PVA and CS not only improves the properties of the polymer system, but also shows the potential for an excellent synergy of biological activities of the two materials. This type of composite material utilizes the water-soluble advantage of poly(vinyl alcohol) for drug delivery and the advantage of CS in the targeting of the acidic and negatively charged areas of diseased cell types.

Usually, studies on these nanofibers were carried out with the use of acetic acid aqueous solvents [30,31,32,33,34]. In practice, most drugs are organic compounds which are soluble in less polar solvents [35], so a decrease of the polarity of the solvent system is more likely to incorporate a higher amount of the drug. For instance, methotrexate has a limited bioavailability due to its water solubility of only 0.01 mg/mL at 20 °C, despite its well-known anti-cancer, anti-rheumatic, dermatological, antimetabolite, and immunosuppressive properties [36]. Melphalan is used for treating various cancers, but its poor solubility in water (0.1 g/mL at 25 °C) and variable bioavailability make it a controversial treatment option [37]. Several other anti-cancer medications, including azathioprine, which has a water solubility of 0.01% (wt./wt.) [38]; 6-mercaptopurine (0.135 mg/mL) [35]; bicalutamide (5 mg/L) [39]; flutamide (practically insoluble in water in the pH range of 1–6) [40]; and others, also have this limitation. Additionally, recently, we demonstrated that the PVA-CS solution in acidic aqueous solution has quite high electrical conductivity, leading to the fabrication of nanofibers, which also requires more precise electrospinning parameters [41,42].

Ethanol is a volatile, inexpensive, readily available, essentially non-toxic solvent that can be used to dissolve a wide variety of organic compounds [43,44,45,46,47,48,49,50,51,52].Therefore, the addition of ethanol to polymer solutions has great potential to improve the integration of organic compounds in general and drug integration in particular, especially ethanol-soluble compounds, into composite systems.

To find the optimal conditions for the fabrication of drug-integrated nanofibers, we investigated the effect of ethanol in an acetic acid solution system on the electrospinning process and properties of PVA-CS nanofibers. The effect of the solvents’ ratio on the chemical composition, crystal structure, and thermomechanical properties of PVA-CS nanofibers was also investigated.

## 2. Materials and Methods

### 2.1. Materials

Poly(vinyl alcohol) with a molecular weight of 75 kDa was purchased from “LenReaktiv” company, Saint Petersburg, Russian Federation, and CS with a molecular weight of 200 kDa and a deacetylation degree of 83% (TU 9289-067-00472124-03) from “Bioprogress” Limited Liability Company, Biokombinat village, Russian Federation, was utilized. Acetic acid 99.5%, ethanol 95%, and distilled water were used as the solvents.

### 2.2. Optical Properties of Polymer Solutions

To study the optical density of polymer solutions, a 2150-UV spectrophotometer (UNICO, Dayton, OH, USA) with a wavelength range of 200–1000 nm was used.

A turbidity meter 2100P (HACH, Düsseldorf, Germany) was used to investigate the turbidity of polymer solutions. The accuracy is ±2%, the resolution is 0.01 FNU, and the reproducibility is ±1%.

### 2.3. Rheological Properties

The pH of the electrospun solutions was determined using a S213 SevenCompact Duo pH/conductivity meter (Mettler Toledo, Greifensee, Switzerland) with the accuracy of 0.002.

The dynamic viscosity of polymer solutions was determined using an MCR 502 rheometer (Anton Paar, Graz, Austria) with a cylinder-cup measuring system. Shear rate ranging from 0.1 to 500 s^−1^ and shear stress was measured at 25 °C.

The conductivity of the polymer solution was measured using the WTW inoLab Cond 7110 conductivity meter with the WTW TetraCon 325 (inoLab, Ankara, Turkey) sensor and S213 SevenCompact Duo pH/conductivity meter.

### 2.4. Electrospinning Technique

To investigate the fibers’ fabrication parameters, an electrospinning system, NANON-01A (MECC Co., Ltd., Fukuoka, Japan), was used. The electrospinning was carried out at a temperature of 28.0 ± 2.0 °C and a relative humidity of 21 ± 3%. The following technological parameters were investigated to facilitate the electrospinning process: the voltage ranges from 16 to 30 kV; the feed rate ranges from 0.1 to 0.4 mL/h; the needle to collector distances ranges from 120 to 150 mm; the horizontal speed—10 mm/s; 16G steel needle; and 150 mm × 200 mm (L × B) stainless steel receiver plate.

The rotating drum of the device at 500 rpm was used to obtain the nanofiber matrix for mechanical property investigations.

### 2.5. Morphology and Diameters of Nanofibers

The preliminary characteristics, morphologies, and diameters of PVA electrospinning fibers were determined using the Olympus STM6 (OLYMPUS Corporation, Tokyo, Japan) measuring optical microscope. The differential interference contrast (DIC) technique was used to emphasize the color and contrast of the fibers. The microphotograph program ImageJ ver. 1.54f 29 June 2023 (National Institutes of Health, Bethesda, MD, USA) was used for the analysis and measurement of the electrospun nanofiber diameter.

### 2.6. Fourier-Transform Infrared (FTIR) Spectroscopy

A Tensor 37 Fourier-transform infrared spectrometer (Bruker, Bremen, Germany) equipped with an attenuated total reflection module (ATR), MIRacle (Pike technologies, Madison, WI, USA) was used to obtain the samples’ infrared absorption spectra. The investigated spectral range was between 4000 and 500 cm^−1^.

### 2.7. X-Ray Diffraction (XRD) Analysis

At room temperature, wide-angle X-ray diffraction (XRD) profiles of polymer powders and nanofibers were obtained using a DRON-8 X-ray unit in a slit configuration with a BSV-29 sharp confocal tube with a copper anode and a NaI (Tl) irradiation probe and a β-filter (Ni). XRD was performed on flat surface samples placed on glass slides. The 2θ range of the samples was 10–60°. The average crystallite size was calculated using the Scherrer formula:

τ = K λ/β cos θ

where τ is average crystallite size, K = shape factor (0.98 rad), λ is X-ray wavelength (1.54 Å), β is the line broadening at half the maximum intensity (FWHM), and θ is Bragg angle. The normalized area of the diffraction peaks was used to calculate the degree of crystallinity [53].

### 2.8. Differential Scanning Calorimetry (DSC) Analysis

Differential scanning calorimetry (DSC) was performed using a DSC 204 F1 Phoenix instrument (Netzsch, Selb, Germany). The experiments were carried out in nitrogen-filled closed aluminum crucibles (protective gas flow rate of 80 mL/min; working gas flow rate of 30 mL/min). Approximately 2 mg of samples was heated and cooled at a rate of 10 K/min in a temperature range of up to 300 °C. Prior to the analysis, samples were heated from room temperature to 150 °C to remove the adsorbed moisture. After 5 min at 150 °C, samples were cooled to −30 °C and then analyzed in the −30 °C to +300 °C temperature range. The comparison cell consists of air.

The heat scan was used to calculate the samples’ degree of crystallinity (χc) using the following equation:

χc = (∆H/m ∆H_o_) × 100

where m is the mass of PVA, ∆H is the melting enthalpy of the sample, and ∆H_o_ is the fusion enthalpy of 100% crystalline PVA, which has been reported to be 150 J/g [54].

### 2.9. Thermogravimetric Analysis (TGA)

The thermogravimetric properties of the initial polymers and the resulting nanofibers were investigated using a TG 209 F1 Libra vacuum-tight microthermal balance (Netzsch, Selb, Germany). The temperature range was 25 °C to 900 °C, the heating rate was 10 °C/min, the nitrogen gas atmosphere flow rate was 40 mL/min, and the measuring cup material was Al_2_O_3_.

### 2.10. Tensile Property

The Instron 5943 tensile testing machine was used to investigate the tensile properties of samples (Instron, Norwood, MA, USA). The test was carried out at room temperature and at a speed of 50 mm/min in accordance with the ISO 527-3 standard [55].

### 2.11. Statistical Analysis

OriginPro 2019b was used to investigate the diameter distribution of materials and nanofibers from micrographs (OriginLab Corporation, Northampton, MA, USA).

To determine the lattice parameters of PVA powder, CS powder, and nanofibers, the DSC and XRD data were analyzed using OriginPro 2018 (SR1 v9.510195) and X’Pert Highscore software (PANalytical, 2009), as well as the DICVOL04 indexing program.

## 3. Results and Discussion

In this study, all concentrations and ratios were calculated as mass percent % (wt/wt) concentrations.

In our recent publication, we described that the best mixture solution composition for the PVA-CS nanofiber fabrication from the same polymer components using a CH_3_COOH-H_2_O solvent system was found to be 4% PVA, 3% CS, 60% CH_3_COOH, and 33% H_2_O [42]. As a result, in this investigation, the polymer and water ratio was maintained while adjusting the acetic acid/ethanol ratio to achieve a total concentration of 60%.

### 3.1. Effect of Ethanol-Acetic Acid Ratio on Optical Properties of PVA-CS Solution

**Figure 1:**
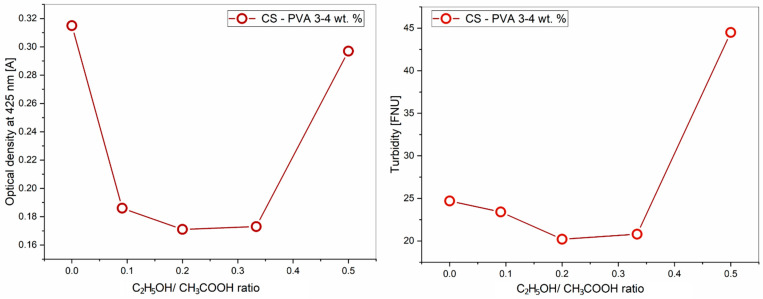
Effect of the ethanol-acetic acid ratio on the optical density and turbidity of the PVA-CS solution.

An aqueous solution containing 4% PVA, 3% CS, and a mixture of ethanol and acetic acid solvents in different ratios was prepared, with a total organic solvent content of 60%. The results of the turbidity and optical density studies of the obtained solutions at a wavelength of 425 nm are presented in Table 1 and Figure 1.

The effects when the ethanol concentration in the solution is increased and the concentration of acetic acid is decreased include the reduction of the acidity and polarity of the PVA-CS solution, as well as the replacement of acetic acid with ethanol in hydrogen bonding with the polymers.

The results presented in Table 1 and Figure 1 demonstrate that although the concentration of each polymer and the concentration of water remained constant, the absorbance (*A*) and turbidity of the solution changed when the ethanol-acetic acid ratio was altered.

According to the Mie theory, the absorbance (*A*) of a solution of identical particles at a particular wavelength (*λ*) of light is described by Formula (1):

(1) $$A=\left(\frac{I}{I_{0}}\right)\;$$

where *I*_0_ is the incident light intensity and *I* is the light intensity after the passing through the solution. By using the extinction cross-section *σ*, path length *l*, and number density *N*, the relationship between *I* and *I*_0_ (Formula (2)) is described in publication [56]:

(2) $$I=I_{0}e^{-\sigma lN}$$

The absorbance (*A*) can be represented by Formula (3):

(3) $$A=\frac{\sigma lN}{ln10}$$

For spherical particles, the extinction cross-section (*σ*) is related to the extinction efficiency (*Q_ext_*) by *σ = πR*^2^*Q_ext_*, where *R* is the spherical particle’s radius [57]. As a result, the absorbance (*A*) can be calculated using Formula (4):

(4) $$A=\frac{\pi R^{2}Q_{ext}lN}{ln10}$$

A solution model with a particle size distribution’s actual absorption spectrum is the total of the weighted sum of the absorption spectra of all of the particle sizes, as shown in Equation (5):

(5) $$A=\sum_{n=1}^{i}W_{i}A_{i}$$

where *n* is total particle sizes in the distribution and *W_i_* and *A_i_* are the weight and absorbance of ith particle size, respectively.

Equation (6) can be obtained by the combination of Equations (4) and (5) as follows:

(6) $$A=\frac{\pi N}{ln10}\sum_{n=1}^{i}R_{i}^{2}Q_{ext,i}W_{i}$$

And number density *N* denotes the total number of particles in the solution per volume unit [58].

This means that an increase in the ethanol concentration and a decrease in the acetic acid concentration significantly affect the size of polymer nanoparticle suspensions as well as their solubility. Acetic acid is a weak electrolyte that can disrupt the long-range intramolecular hydrogen bonds of polymers. These bonds in solution are converted into intermolecular hydrogen bonds, increasing the entanglement between them, leading to an increase in the viscosity of the solution as well as the size of the macrochains in the system [59,60,61]. Ethanol is a non-electrolyte and is not capable of changing the intermolecular and intramolecular bonds in polymer solutions [62,63]. The replacement of acetic acid with ethanol reduced the number of interhydrogen bonds in the solution and lessened the size and mass of the nanoparticles. It is the diminishment of the nanoparticle size that causes a decrease in the optical density and turbidity of the solution at ethanol concentrations below 20%.

When the ethanol concentration reached 20% and the concentration of acetic acid decreased to 40%, the pH and polarity of the solution were insufficient for the complete polycation CS formation. This leads to a decrease in the CS solubility and the onset of phase separation. As a result, both the optical density and the turbidity of the solution sharply increased. When the ratio of ethanol-acetic acid reaches 25/35, the PVA-CS solution is no longer formed.

The change in the state of polymers under the influence of the ethanol-acetic acid ratio significantly affects the rheological properties and especially the viscosity of PVA-CS solutions.

### 3.2. Effect of Ethanol-Acetic Acid Ratio on Rheological Properties of PVA-CS Solution

**Figure 2:**
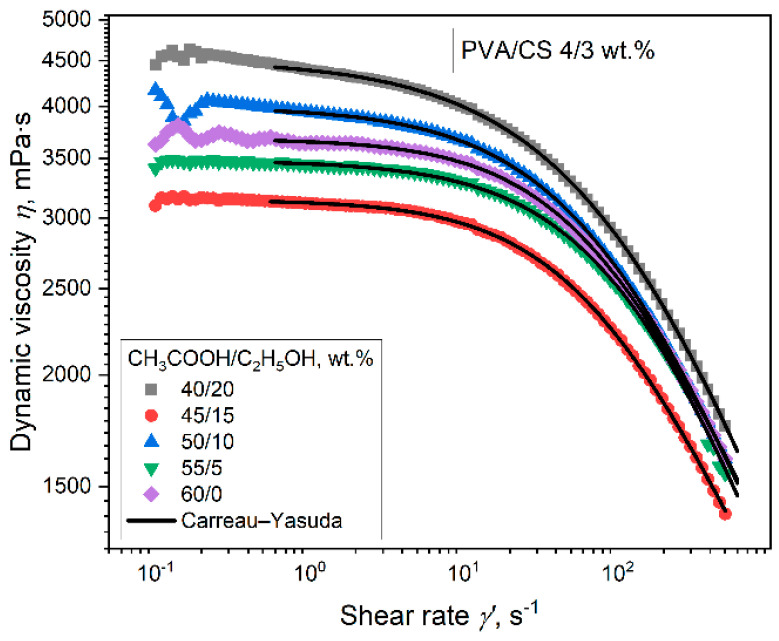
Graphs of the dependence of shear rates on the dynamic viscosity of PVA-CS solutions at the different ratios of ethanol-acetic acid.

Aqueous solutions containing 4% PVA, 3% CS, and a mixture of ethanol and acetic acid solvents in different ratios (the total concentration of the organic solvents was 60%) were prepared. The rheological properties of these solutions were then investigated (Figure 2).

The flow curve of a PVA-CS 4–3 solution under the influence of different ratios of ethanol-acetic acid also shows the character of a complex flow with three stages:

(1) the region of shear rates up to 1.5 s^−1^—destruction of the initial structure of the solution; (2) the region of shear rates from 1.5 to 10 s^−1^, corresponding to Newton’s law of viscous friction; and (3) the region of shear rates greater than 10 s^−1^, corresponding to the mechanism of non-Newtonian pseudoplastic flow thinning.

The Carreau-Yasuda model of non-Newtonian liquid flow was applied for a complex description of the flow of the solutions in the second and third ranges [64,65]:

$$\eta (\gamma^{\cdot })\;=\eta_{0}{[1\;+\;{\left(\lambda \gamma^{\cdot }\right)}^{a}]}^{\frac{m-1}{a}}$$

where *η*_0_ is the zero shear viscosity, *λ* is the characteristic time constant, a is the change of flow type, and *m* is the flow (pseudoplasticity) index. In Figure 2, the fitting curves of the Carreau-Yasuda equation are shown as black lines. The parameters of the Carreau–Yasuda equation for the curves (Figure 2) are presented in Table 2.

The values of the coefficient of determination clearly indicate that the model describes the considered shear rate ranges almost perfectly. It can be seen that the values of *η*_0_, which characterize the total viscosity of the solution in the second range of shear rates, change in a complex manner with an increase in the ethanol-acetic acid ratio.

In this case, the flow rate m depends on the ratio of ethanol-acetic acid. The flow index m is less than 1, which characterizes the active shear thinning of the system [64].

The complex variation in the values of viscosity η_0_ and the flow index m of PVA-CS solutions under the influence of the ethanol-acetic acid ratio is associated with a modification in the structure and state of macromolecules in the solution. Alterations in the size of nanoparticles, particle density, and polymer solubility, in addition to adaptations of the values of optical density and turbidity, obviously had a significant effect on the rheological properties of PVA-CS (the ratio 4–3) solution.

**Figure 3:**
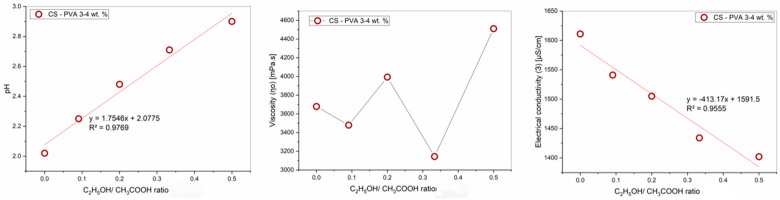
Graphs of the dependence of the rheological properties of the PVA-CS solution on the ethanol-acetic acid ratios.

The influence of the ethanol-acetic acid ratios on the pH, viscosity, and electrical conductivity of the PVA-CS 4–3 solutions is presented in Table 3 and Figure 3.

The rheological properties of the solution vary greatly depending on the ratio of ethanol and acetic acid in the solution, where the pH value increases with a decrease in the acetic acid concentration, and the viscosity and conductivity values change inconsistently.

As mentioned above, the heterogeneous increase in viscosity *η*_0_ is caused by two opposing trends in viscosity variations when the acetic acid concentration decreases and ethanol concentration increases. The first one is that when the density of hydrogen bonds between acetic acid molecules and polymers is reduced, the size of macromolecule blocks and viscosity decreases. The second tendency is the decrease in CS solubility due to an insufficient pH value for the CS polycation formation, which enhances the polymer’s intermolecular stickiness and viscosity. As a result, the viscosity of the PVA-CS solutions changed unevenly.

The electrical conductivity depends on the density of free ions and the viscosity of the solution and therefore also changes heterogeneously. However, in general, the conductivity of the solution decreases with an increase in the ethanol-acetic acid ratio.

The change of rheological properties plays an important role in the nanofiber fabrication by electrospinning. In the previous study on the fabrication of PVA-CS nanofibers from a solution of 4% PVA and 3% CS without ethanol, the obtained nanofiber diameter was 326 ± 62 nm at the optimal needle-collector distance of 140 mm, feed rate of 0.1 mL/h, and voltage of 27 kV [42]. In this work, we investigated the effect of the addition of ethanol to the electrospinning solution on nanofiber fabrication, and the properties of the obtained PVA CS nanofibers. The electrospinning parameters have been changed to find optimal conditions for fiber fabrication.

### 3.3. Effect of Ethanol-Acetic Acid Ratio on PVA-CS Nanofiber Fabrication

#### 3.3.1. The PVA-CS Nanofiber Fabrication Ability and the Dependence on the Solvent Ratio

Solutions containing 4% PVA, 3% CS, and 60% acetic acid and ethanol mixtures were electrospun. The influence of the technological parameters, including the distance between the needle tip and the collector being varied from 100 to 150 mm, the voltage being changed from 16 to 30 kV, and the flow rate being adjusted from 0.1–0.2 mL/h on the nanofibers fabrication, is presented in Table 4.

Thus, the PVA-CS nanofiber fabrication ability is the greatest when the ethanol-acetic acid ratio is 15–45.

#### 3.3.2. Intermolecular Interactions for the PVA-CS Nanofiber Fabrication

The impact of intermolecular interactions between polymers and solvents as well as between polymers themselves on the acquired properties of polymer nanofibers is one of the most significant concerns related to the electrospinning process.

Based on the advancement of the Scatchard hypothesis, Joel H. Hildebrand suggested using the square root of the cohesive energy density as a numerical value for the determination of a solvent’s solvency.

(7) $$\partial =\sqrt{c}={\left[\frac{∆H-RT}{V_{m}}\right]}^{\frac{1}{2}}\;\;\;\;\;({MPa}^{1/2})$$

where *c* is the cohesive energy density, Δ*H* is the heat of vaporization, *R* is the gas constant, *T* is the temperature, and *V_m_* is the molar volume [66,67].

The Hildebrand value for a solvent mixture can be determined by averaging the Hildebrand values of each solvent by volume. The overall Hildebrand value is divided into three parts by Hansen parameters: the dispersion, polar, and hydrogen components, with the dispersion component counting for the majority of these interactions.

(8) $$\partial_{t}^{2}=\partial_{d}^{2}+\partial_{p}^{2}+\partial_{h}^{2}$$

where $\partial_{t}^{2}$ is the total Hildebrand parameter, $\partial_{d}^{2}\;$ is the dispersion component, $\partial_{p}^{2}$ is the polar component, and $\partial_{h}^{2}$ is the hydrogen bonding component. The Hansen parameters for the solvents utilized in this investigation are listed in Table 5 [66,67].

The Teas graph is based on the assumption that all materials have the same Hildebrand value. According to this, solubility behavior is determined by the proportions of the three component forces (dispersion force *f_d_*, polar force *f_p_*, and hydrogen bonding force *f_h_*) that contribute to the total Hildebrand value. Teas parameters represent the percentage contribution of each Hansen parameter to the total Hildebrand value.

(9) $$f_{d}=\frac{\partial_{d}}{\partial_{d}+\partial_{p}+\partial_{h}};\;f_{p}=\frac{\partial_{p}}{\partial_{d}+\partial_{p}+\partial_{h}};\;f_{h}=\frac{\partial_{h}}{\partial_{d}+\partial_{p}+\partial_{h}}$$

(10) $$f_{d}+f_{p}+f_{h}=100$$

The fractional solubility parameters for the solvents used in this investigation are listed in Table 6 [66].

**Figure 4:**
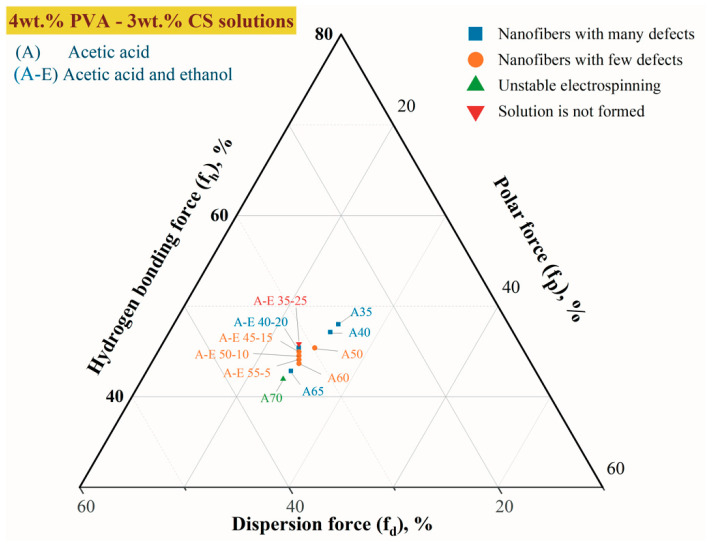
The Teas plot for PVA-CS electrospinning solutions with CH_3_COOH-H_2_O and CH_3_COOH-C_2_H_5_OH-H_2_O as the co-solvent system.

The solubility parameters of the components approach each other as the molar enthalpy of mixing (Δ*H*) approaches zero. Figure 4 depicts a Teas diagram that accounts for each component’s contribution (assuming that their sum equals 100%) and is based on the presumption that all materials are equal.

For every part of the experimental combination, dispersion forces (*f_d_*), polar forces (*f_p_*), and hydrogen bond forces (*f_h_*) were calculated. These fiber formation parameters are presented in Table 7.

**Figure 5:**
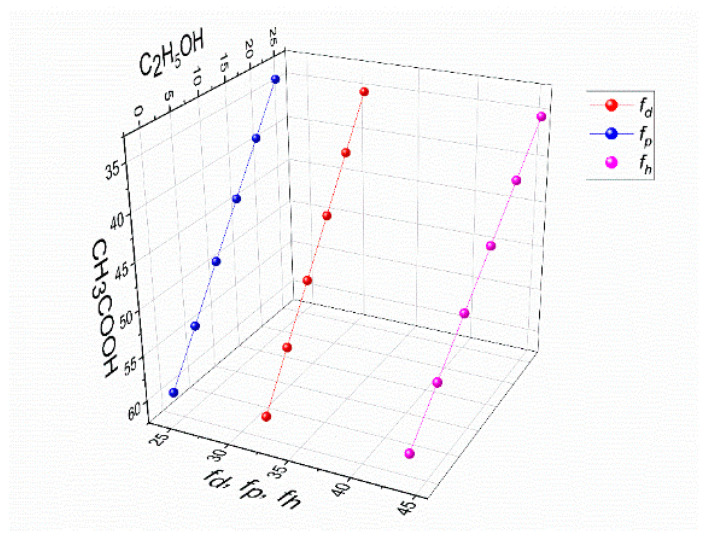
The values of polar interactions *f_h_*, *f_p_*, and *f_d_* for a mixture of solvents, CH_3_COOH-C_2_H_5_OH-H_2_O, in the solution of 4% PVA–3% CS.

In our previous work, the data on the influence of acetic acid on the solubility parameters of the 4% PVA–3% CS solutions were reported [42]. To better illustrate the differences in effects between the two solvent systems with and without ethanol, Figure 5 reconstructs the Teas diagram for both co-solvent systems.

Given that water makes up 33% of the mixtures in this experiment, it was possible to create a three-dimensional graph by plotting the concentrations of solely CH_3_COOH and C_2_H_5_OH along the *X* and *Y* axes, while the values of *f_d_*, *f_p_*, and *f_h_* were drawn along the *Z* axis (Figure 5).

Compared to the solution without ethanol, it is evident that the addition of ethanol to PVA-CS-CH_3_COOH solutions has an effect on the stabilization of the parameters of 4% PVA–3% CS solutions.

Thus, it can be concluded that binary, trinary, or other multi-component solutions with a “*f_h_ − f_p_*” value between 19.5 and 21.5% are appropriate for the use in the electrospinning process for PVA-CS nanofiber fabrication.

#### 3.3.3. Morphology of PVA-CS Nanofibers

**Figure 6:**
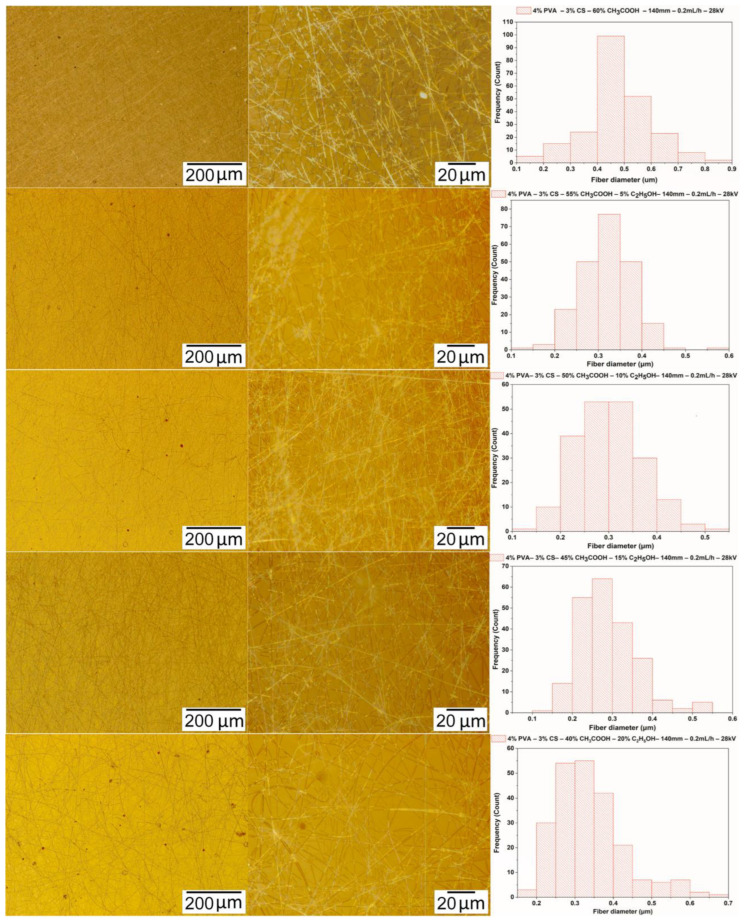
Microscopic images at 100× and 1000× and the diameter distributions of PVA-CS nanofibers obtained from solutions of 4% PVA, 3% CS, and different concentration of acetic acid and ethanol (electrospinning parameters fixed at a collector—needle distance of 140 mm, a feed rate of 0.2 mL/h, and a voltage of 28 kV).

Micrographs of nanofibers obtained from 4% PVA–3% CS-ethanol-acetic acid solutions with the electrospinning parameters fixed at a needle-collector distance of 140 mm, a voltage of 28 kV, and a feed rate of 0.2 mL/h were used to investigate the effect of ethanol-acetic acid ratios on the morphology and the diameter of PVA-CS nanofibers (Figure 6 and Table 8).

From the data presented in Table 8 and Figure 6, it can be concluded that the addition of ethanol to the electrospinning solution clearly altered the fabrication ability of PVA-CS nanofibers as well as their morphology and diameter. The results of microimage analysis and diameters of nanofibers obtained from solutions with different concentrations of ethanol-acetic acid revealed that the solution with ethanol-acetic acid (at a ratio of 15–45) has the lowest viscosity and is also the solution for high nanofiber fabrication capability with a better morphology and smaller diameter.

The observation of nanofiber formation during the electrospinning as well as the results of the analysis of the micrographs and the diameter distribution of the nanofibers from solutions with the different ethanol-acetic acid ratios showed that the solution of 4% PVA, 3% CS, 15% ethanol, and 45% acetic acid was the optimal one for PVA-CS nanofiber fabrication. As a result, further investigation of the electrospinning parameters is required to determine the optimal technological parameters.

### 3.4. Effect of Ethanol-Acetic Acid Ratio on Electrospinning Parameters

#### 3.4.1. Parameter of the Distance Between the Needle Tip and the Collector

**Figure 7:**
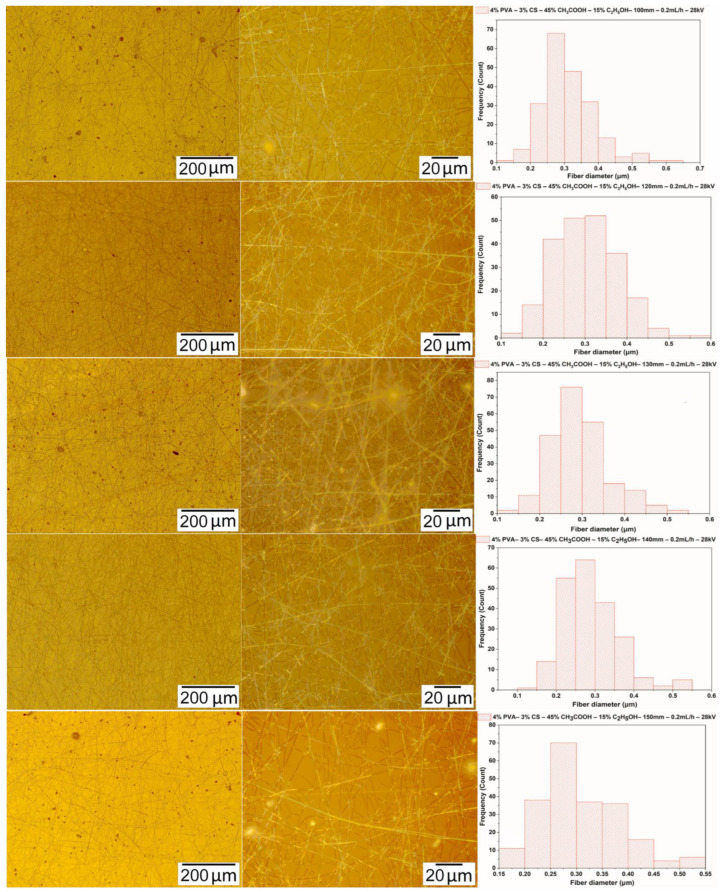
Microscopic images at 100× and 1000× and the diameter distributions of PVA-CS nanofibers obtained from the solution of PVA-CS (the ratio being 4–3) with an ethanol-acetic acid solvent ratio of 15–45 at a feed rate of 0.2 mL/h, a voltage of 28 kV, and with variation of the needle-collector distance.

The electrospinning was performed with a solution of PVA-CS (the ratio 4–3) with an ethanol-acetic acid ratio of 15–45, with the distance between the needle and the collector varying from 100 to 150 mm and the voltage and the speed fixed at 28 kV and 0.2 mL/h, respectively. Figure 7 and Table 9 present the data on the form and diameter distribution of PVA-CS nanofibers.

Figure 7 and Table 9 show microimages and statistics on fiber diameter distribution. These data demonstrate that the optimal distance between the needle and the collector for the electrospinning is 140 mm. At this parameter, the fibrous film has the least number of defects and the fiber diameter is also the smallest.

#### 3.4.2. Parameter of the Feed Rate

**Figure 8:**
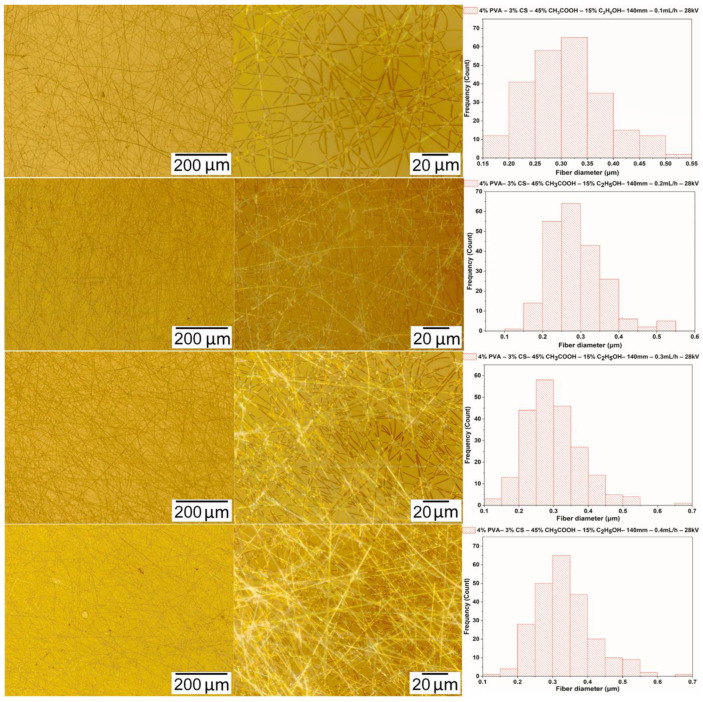
Microscopic images at 100× and 1000× and diameter distributions of PVA-CS nanofibers obtained from the solution of PVA-CS (the ratio 4–3) with an ethanol-acetic acid solvent ratio of 15–45 at a needle-collector distance of 140 mm, a voltage of 28 kV, and a variation of the feed rate from 0.1 to 0.4 mL/h.

The solution of PVA-CS (the ratio being 4–3) with the ethanol-acetic acid solvent (the ratio being 15–45) was electrospun with the voltage and the needle-collector distance fixed at 28 kV and 140 mm, respectively, and the rate was adjusted from 0.1 mL/h to 0.4 mL/h. Microimages and fiber diameter distribution statistics are shown in Figure 8 and in Table 10.

Thus, at a needle-collector distance parameter of 140 mm, a feed rate of 0.2 mL/h, and a voltage of 28 kV, the PVA-CS nanofibers obtained have the smallest diameter of 285 ± 65 nm. At a feed rate of 0.3 mL/h, although the PVA-CS nanofibers have fewer defects, the fiber diameter is larger.

#### 3.4.3. Parameters of the Voltage

**Figure 9:**
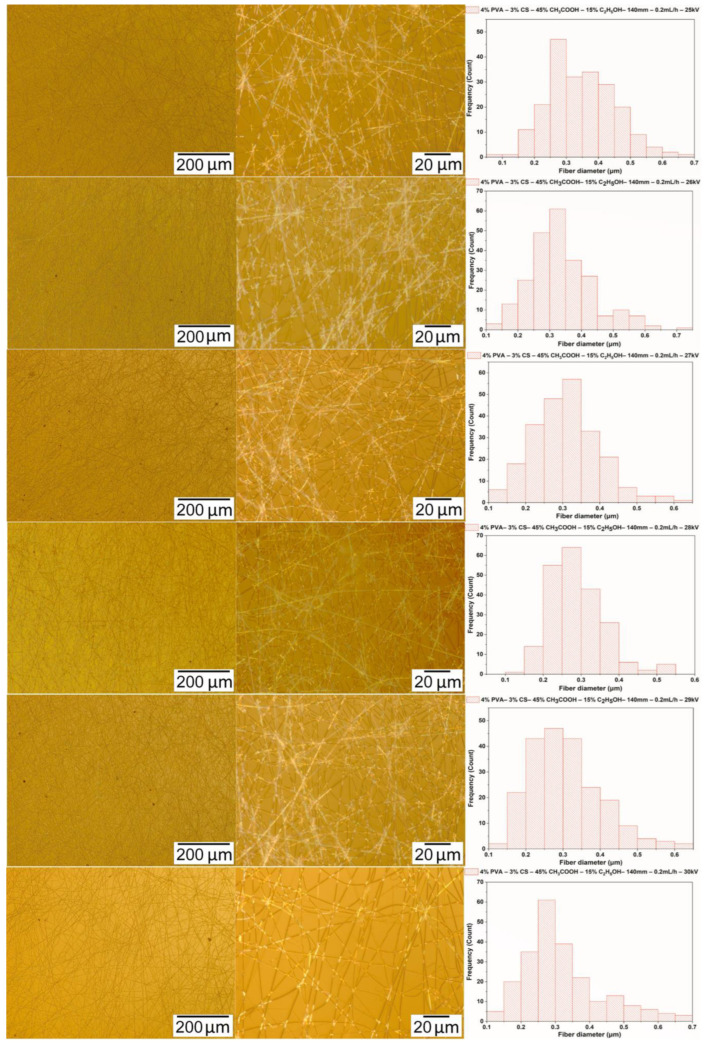
Microscopic images at 100× and 1000× and diameter distributions of PVA-CS nanofibers obtained from the solution of PVA-CS (the ratio 4–3) with an ethanol-acetic acid solvent ratio of 15–45 at a needle-collector distance of 140 mm, a feed rate of 0.3 mL/h, and a variation in voltage from 25 to 30 kV.

The solution of PVA-CS (the ratio being 4–3) with an ethanol-acetic acid solvent ratio of 15–45 was subjected to electrospinning with a fixed distance between the needle and the collector of 140 mm, a flow rate set at 0.2 mL/h, and a voltage change between 25 kV and 30 kV. Microscopic images and fiber diameter distribution statistics are shown in Figure 9 and in Table 11.

The morphological analysis and diameter distribution of PVA-CS nanofibers revealed that at 28 kV, the obtained fibers had the smallest diameter with the fewest defects. The optimal technological parameters for the electrospinning include a needle-collector distance of 140 mm, a feed rate of 0.2 mL/h, and a voltage of 28 kV. The obtained PVA-CS nanofibers have a diameter of 285 ± 65 nm.

The fabrication of PVA-CS nanofibers without ethanol leads to the nanofiber diameter 326 ± 62 nm, and the optimal feed rate was only 0.1 mL/h. When compared to the acetic acid, the mixed solvent of 45% acetic acid and 15% ethanol not only improved the morphology and diameter of PVA-CS nanofibers, but also doubled or tripled the nanofiber fabrication yield (at a feed rate of 0.3 mL/h, the diameter was 300 ± 79 nm).

### 3.5. Fourier-Transform Infrared (FTIR) Spectroscopy

In our previous study [42], it was confirmed that acetic acid totally separates from the nanofiber matrix and has no effect on the polymeric components of PVA-CS nanofibers. Infrared spectra of nanofibers fabricated from the solutions of PVA-CS and ethanol-acetic acid in different ratios were taken in order to analyze the effect of ethanol on the chemical bond formation in the system of PVA-CS nanofibers.

**Figure 10:**
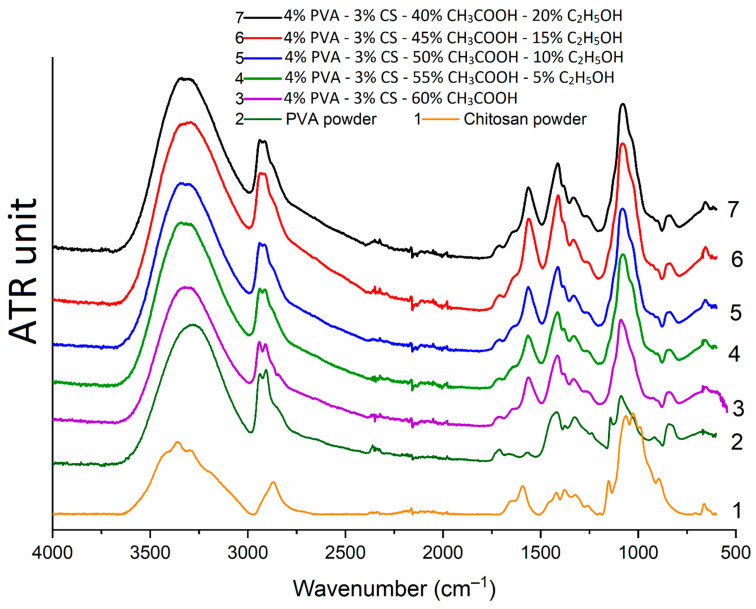
Infrared spectra of PVA powder, chitosan powder, and PVA-CS nanofibers.

Infrared spectra of PVA-CS nanofibers (Figure 10) demonstrate that they are the same. The peak positions did not change, indicating that no new chemical bonds were formed when ethanol was added to the electrospinning solution system. Thus, it is evident that during the electrospinning, ethanol and acetic acid were totally separated from the PVA-CS nanofiber mat.

In the case of the PVA-CS nanofibers, the IR spectra of the nanofiber systems become simpler and closely resemble that of pure PVA. The oscillations of the C–H alkyl bond in the CS molecule at 2869 cm^−1^ [68] take on a shoulder shape when combined with the asymmetric and symmetric stretching bands of the CH_2_ groups in the PVA molecule at 2940 cm^−1^ and 2910 cm^−1^ [69,70]. In the PVA-CS nanofibers, the bands of each polymer in the 1590–890 cm^−1^ region become simpler, wider, less multiple, and shift into the space between bands. These is evidence that PVA and CS formed the hydrogen bonds in the PVA-CS nanofiber system.

There was a combination of bands at 3279 cm^−1^ (of -OH bonds in PVA molecule), 3300 cm^−1^, and 3362 cm^−1^ (of -OH and -NH bonds in CS molecule) to form a broad and high-intensity band in the region of 3000–3600 cm^−1^ common to all -OH and -NH groups in the polymer system [68,69,70,71]. The 896 cm^−1^ band of the -OH out-of-plane vibrations and the 690 cm^−1^ band of the N–H twist vibrations related to ring stretching in the IR spectrum of CS disappeared in the nanofiber spectra, demonstrating that the mobilities of the -OH and -NH_2_ groups of CS disappeared [72,73,74]. The disappearance of these mobilities, together with the broadening of the absorption region in the range 3000–3600 cm^−1^, suggests that a significant number of hydrogen bonds formed between the PVA and CS molecules. Similar conclusions were reached in previous publications [70,71].

### 3.6. X-Ray Diffraction (XRD) Analysis

**Figure 11:**
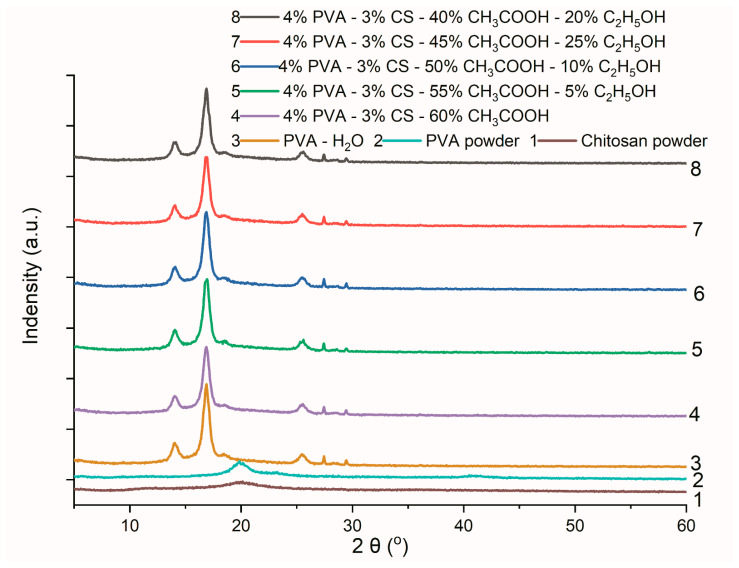
X-ray diffraction data of PVA powder, CS powder, and PVA nanofibers from an aqueous solution and PVA-CS nanofibers from an aqueous solution with C_2_H_5_OH and CH_3_COOH.

The XRD spectral data are shown in Figure 11, and the results of the crystal lattice parameters of the obtained nanofiber systems are presented in Table 12.

Overall, compared to the polymer powders, the XRD spectra of the PVA and PVA-CS nanofibers have stronger and more noticeable peaks. As PVA was converted from a powder to nanofiber, the position and intensity of its peaks altered significantly. The PVA-CS nanofibers’ XRD peak intensities varied slightly from those of the PVA nanofibers and from each other, indicating that solvent factors in addition to the presence of CS and the powder-to-nanofiber transitions affect the crystal structure of these nanofibers.

The results (Table 12) revealed that the increase in the ethanol concentration and the decrease in the acetic acid concentration in the initial electrospinning solution caused a unit cell volume reduction in the PVA-CS nanofibers. However, in the absence of ethanol, the volume of the lattice unit cell is the smallest.

The crystallinity of the nanofiber systems as well as the axial lengths of the irregularly varied crystal cells can be derived from the irregular variation of the viscosity and electrical conductivity of the original solutions.

The variation in crystal structure implies that the ethanol and acetic acid components in the initial electrospun solution also have an impact on the mechanical and thermal properties of the produced PVA-CS nanofiber systems.

### 3.7. Differential Scanning Calorimetry (DSC) Analysis

**Figure 12:**
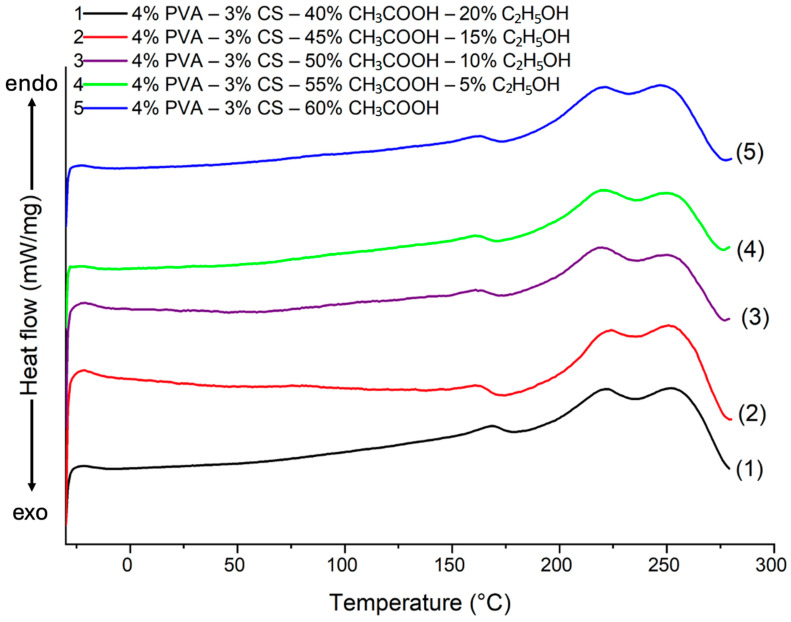
DSC heating curve of PVA-CS nanofibers at different ethanol-acetic acid ratios.

DSC curves of the electrospun PVA-CS nanofibers at different solvent ratios are shown in Figure 12 and in Table 11.

The enthalpy change (ΔH), crystallinity (χ), melting temperature (T_m_), and glass transition temperature (T_g_) results for the PVA-CS nanofibers at different ethanol-acetic acid ratios in electrospinning solutions are presented in Table 13.

The DSC heating curves of PVA-CS nanofibers at various ethanol-acetic acid ratios are slightly different. Specifically, as the percentage of ethanol increases, both the crystallization temperature and the melting point increase slightly. This indicates that the different solvent ratios had an effect on the linking intensity of the polymers during the electrospinning process.

There is correlation between both the results presented in Table 13 and those in Table 1 and Figure 1. The change of the specific enthalpy of PVA on the DSC curve of PVA-CS nanofibers was decreased when the optical density (*A*) and turbidity of the solution dropped (at acetic acid-ethanol ratios of 55–5, 50–10, and 45–15). This suggests that the aggregation state of polymer macromolecules in the initial solution influences polymer crystallization during electrospinning.

The reduction of ΔH_PVA_ by about 20–30% while ΔH_CS_ increased 2-fold suggests that the enhancement of the ethanol ratio in the initial solution mainly affects the structure of CS macromolecules rather than the PVA macromolecules.

Thus, an alteration of the ethanol-acetic acid ratio in the initial solution results in a change in the polymer’s aggregation state in the initial solution, which leads to an adjustment of the individual crystal composition of each polymer in the structure of the PVA-CS nanofiber system. This results in a modification in the lattice, which affects the overall crystallinity of the system. In general, the increase in the ethanol concentration decreases the volume of the unit cell in the crystal lattice and raises the temperature of crystallization and the melting point of the system.

### 3.8. Thermogravimetric Analysis (TGA)

**Figure 13:**
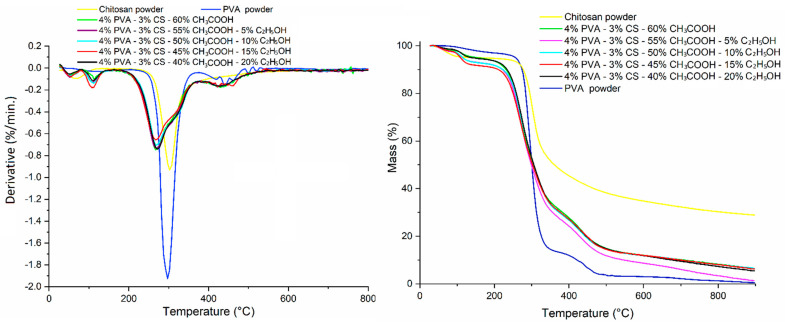
TGA thermogram of PVA powder, CS powder, and PVA-CS nanofibers at different ethanol-acetic acid ratios in initial electrospun solution.

TGA curves for the PVA powder, CS powder, and PVA-CS nanofibers at different solvent ratios are shown in Figure 13. The phases of thermal degradation and weight reduction of these powders and nanofibers are presented in Table 14.

The path of PVA powder and PVA nanofibers goes through three basic stages of thermal degradation, whereas that of CS powder only goes through two. The main stage of the CS powder decomposes at a higher temperature than the other samples, demonstrating that the CS molecules are more heat resistant due to their intricate structure. However, as a result of that, almost all CS bonds break down during this stage.

PVA-CS nanofibers’ thermal degradation process includes the thermal decomposition of the two polymers. This also confirms that the solvents were totally removed from the nanofiber system during the electrospinning process. The decomposition temperatures of these nanofibers, on the other hand, were all lower than those of pure polymers, indicating that their complexes were formed by weak bonds.

Thus, the change in the ethanol-acetic acid ratio has no effect on the thermal decomposition of the PVA-CS nanofibers.

### 3.9. Tensile Property

**Figure 14:**
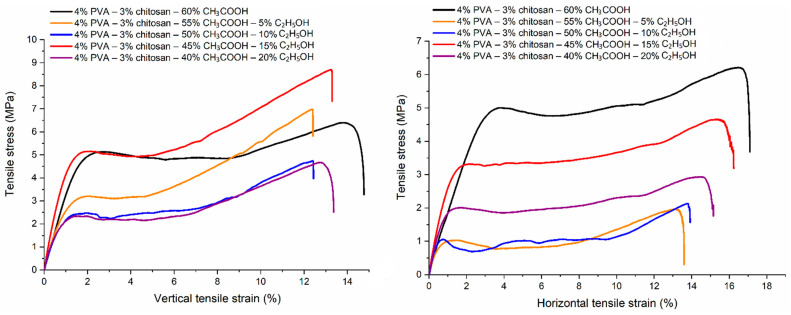
The deformation of PVA-CS nanofibers according to the ethanol-acetic acid ratios in the electrospun solutions.

**Figure 15:**
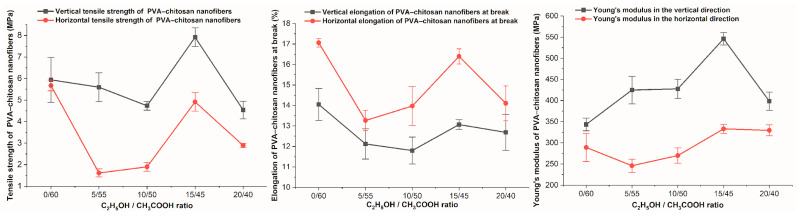
Changes in tensile properties of PVA-CS nanofibers according to ethanol-acetic acid ratios in the electrospun solutions.

The vertical and horizontal tensile properties of PVA-CS nanofiber matrices prepared from 4% PVA, 3% CS, and different ethanol-acetic acid ratios were investigated (Table 15, Figure 14 and Figure 15).

Each sample has a vertical tensile strength and Young’s modulus that are greater than their horizontal counterparts, as well as a vertical elongation at break that is smaller than the horizontal elongation.

Similar to the change in the rheological properties of PVA-CS solutions in acetic acid and ethanol solutions, it is evident that there is no discernible pattern in the variation of the samples’ tensile characteristics.

Compared to the PVA-CS nanofibers from the ethanol-free solution, the PVA-CS nanofibers obtained with an ethanol-acetic acid solution (with a ratio of 15–45), the vertical tensile strength increased by 33%, the horizontal tensile strength decreased by 12%, the elongation at break was reduced by 1%, and the vertical and horizontal Young’s modulus improved by 59% and 15%, respectively.

## 4. Conclusions

For the first time, ethanol was used as the co-solvent in the electrospinning solution of PVA-CS and showed good effects for the improvement of the morphology and fabrication yield of PVA-CS nanofibers.

The use of ethanol and acetic acid demonstrates the effect of shifting the solvent system’s solubility parameters, changing the interaction of the different component forces in the solution and thus influencing the optical and rheological properties of the PVA-CS solution.

To summarize, using an ethanol-acetic acid ratio of 15–45 in the PVA-CS solution resulted in the following outcomes:

- Improved morphology, diameter, and fabrication productivity of PVA-CS nanofibers by 2–3 times due to the reduction of defects, reducing the diameter of the nanofibers from 326 ± 62 nm (0.1 mL/h without ethanol) to 285 ± 65 nm (0.2 mL/h) or 300 ± 79 nm (0.3 mL/h);
- No change in the chemical nature of the resulting system of nanofibers, thereby maintaining the thermal properties of the PVA-CS nanofiber system;
- A change in the structure of the crystal lattice of the nanofibers, which leads to a modification in the mechanical properties of the nanofibers. The PVA-CS nanofibers appear to become stronger via an increase in the vertical tensile strength of 33%, an increase in the vertical and horizontal Young’s moduli of 59% and 15%, and a reduction in the elongation at break of 1%.

Additionally, the use of a multi-component solvent system increases the solubility of small biological active compounds, which allows us to increase the drug amount in the PVA-CS solution.

## Figures and Tables

**Table 1:** Effect of the ethanol-acetic acid ratio on the optical density and turbidity of the PVA-CS solution.

CH_3_COOH/C_2_H_5_OH, %	Optical Density		Turbidity, FNU
	Absorption (A)	Transmission (T), %	
60/0	0.315	48.4	24.7
55/5	0.186	65.2	23.4
50/10	0.171	67.4	20.2
45/15	0.173	67.2	20.8
40/20	0.297	50.5	44.5

**Table 2:** The parameters of the Carreau-Yasuda equation for PVA-CS solutions at the different ratios of ethanol-acetic acid.

CH_3_COOH/C_2_H_5_OH, (%)	*η*_0_, (mPa·s)	*λ* · 10^3^ (s)	*a*	*m*	*R* ^2^
60/0	3678	13.86	0.93	0.611	0.9999
55/5	3479	7.11	0.84	0.507	0.9995
50/10	3993	11.54	0.80	0.553	0.9999
45/15	3143	13.70	0.92	0.617	0.9999
40/20	4511	7.56	0.68	0.485	0.9999

**Table 3:** Effect of ethanol-acetic acid ratio on rheological properties of PVA-CS solution.

C_2_H_5_OH/CH_3_COOH, (%)	pH	Viscosity *η*, (mPa·s)	Electrical Conductivity Ɜ, (μS/cm)
0/60	2.02	2724.3	1611
5/55	2.25	2644.6	1541
10/50	2.48	2796.7	1505
15/45	2.71	2338.4	1434
20/40	2.90	3036.7	1402

**Table 4:** The PVA-CS nanofiber fabrication at different concentrations of ethanol and acetic acid under electrospinning conditions: a needle-collector distance of 100–150 mm, a feed rate of 0.1–0.2 mL/h, and a voltage of 16–30 kV.

Distance (mm)	Feed Rate (mL/h)	C_CH3COOH_ (% *w*/*w*)	C_C2H5OH_ (% *w*/*w*)	Voltage (kV)									
				16	18	20	22	24	26	27	28	29	30
150	0.1	60	0	O	O	o	+	+	+	+	+	+	+
		55	5	O	O	o	+	+	+	+	+	+	+
		50	10	O	O	o	+	+	+	+	+	+	+
		45	15	O	o	+	+	+	+	+	+	+	+
		40	20	O	o	+	+	+	+	+	+	+	+
	0.2	60	0	O	O	O	o	+	+	+	+	+	+
		55	5	O	O	o	o	+	+	+	+	+	+
		50	10	O	O	o	o	+	+	+	+	+	+
		45	15	O	O	o	+	+	+	+	+	+	+
		40	20	O	O	o	+	+	+	+	+	+	+
140	0.1	60	0	O	o	+	+	+	+	+	+	+	+
		55	5	O	O	o	+	+	+	+	+	+	+
		50	10	O	O	o	+	+	+	+	+	+	+
		45	15	O	o	+	+	+	+	+	+	+	+
		40	20	O	o	+	+	+	+	+	+	+	+
	0.2	60	0	O	O	O	o	+	+	+	+	+	+
		55	5	O	O	o	o	+	+	+	+	+	+
		50	10	O	O	o	*	+	+	+	+	+	+
		45	15	O	O	*	+	+	+	+	+	+	+
		40	20	O	O	o	+	+	+	+	+	+	+
120	0.1	60	0	O	o	+	+	+	+	+	+	+	+
		55	5	O	o	+	+	+	+	+	+	+	+
		50	10	O	o	+	+	+	+	+	+	+	+
		45	15	o	+	+	+	+	+	+	+	+	+
		40	20	o	o	+	+	+	+	+	+	+	+
	0.2	60	0	O	o	o	*	*	+	+	+	+	+
		55	5	O	O	o	+	+	+	+	+	+	+
		50	10	O	O	o	+	+	+	+	+	+	+
		45	15	O	o	+	+	+	+	+	+	+	+
		40	20	O	o	o	+	+	+	+	+	+	+
100	0.1	60	0	O	o	+	+	+	+	+	+	+	+
		55	5	O	o	+	+	+	+	+	+	+	+
		50	10	O	o	+	+	+	+	+	+	+	+
		45	15	o	+	+	+	+	+	+	+	+	+
		40	20	o	+	+	+	+	+	+	+	+	+
	0.2	60	0	O	o	+	+	+	+	+	+	+	+
		55	5	O	o	*	+	+	+	+	+	+	+
		50	10	O	o	*	+	+	+	+	+	+	+
		45	15	o	*	+	+	+	+	+	+	+	+
		40	20	o	*	+	+	+	+	+	+	+	+

(+) the formation of fibers; (O) the formation of droplets and a few fibers; (o) the formation of fibers and a few drops; (*) the formation of fibers, but the process is unstable.

**Table 5:** The Hansen parameters for the solvents CH_3_COOH, C_2_H_5_OH, and H_2_O [66,67].

Solvent	*∂_t_*	*∂_d_*	*∂_p_*	*∂_h_*
CH_3_COOH	21.4	14.5	8	13.5
C_2_H_5_OH	26.5	15.8	8.8	19.4
H_2_O	47.8	15.6	16	42.3

**Table 6:** The fraction solubility parameters for the solvents CH_3_COOH, C_2_H_5_OH, and H_2_O [66].

Solvent	100 *f_d_*	100 *f_p_*	100 *f_h_*
CH_3_COOH	40	22	38
C_2_H_5_OH	36	18	46
H_2_O	18	28	54

**Table 7:** The parameters of fractional solubility in the solution of 4% PVA–3% CS in the mixture of the solvents CH_3_COOH-C_2_H_5_OH-H_2_O.

CH_3_COOH wt.%	C_2_H_5_OH wt.%	H_2_O wt.%	100 *f_d_*	100 *f_p_*	100 *f_h_*	*f_h_ − f_p_*	*f_h_ − f_d_*	*f_p_ − f_d_*
60	0	33	32.19	24.13	43.68	19.55	11.48	−8.06
55	5	33	31.98	23.91	44.11	20.19	12.13	−8.06
50	10	33	31.76	23.70	44.54	20.84	12.77	−8.06
45	15	33	31.55	23.48	44.97	21.48	13.42	−8.06
40	20	33	31.33	23.27	45.40	22.13	14.06	−8.06
35	25	33	31.12	23.05	45.83	22.77	14.71	−8.06

**Table 8:** Diameter distributions of electrospun PVA nanofibers obtained from solutions of 4% PVA, 3% CS, and different ratios of acetic acid/ethanol with the fixed electrospinning parameters at a collector—needle distance of 140 mm, a feed rate of 0.2 mL/h, and a voltage of 28 kV.

Diameter (nm)	C_2_H_5_OH/CH_3_COOH Ratio (% *w*/*w*)				
	0/60	5/55	10/50	15/45	20/40
Mean	330	320	301	285	337
Standard deviation	68	59	68	65	91
Min	134	147	110	121	170
Max	587	573	533	592	654

**Table 9:** Diameter distribution of PVA-CS nanofibers obtained from a solution of PVA-CS (the ratio being 4–3) with an ethanol-acetic acid solvent ratio of 15–45 at a feed rate of 0.2 mL/h, a voltage of 28 kV, and with variation of the needle-collector distance.

Diameter (nm)	Needle-Collector Distance (mm)				
	100	120	130	140	150
Mean	313	302	295	285	308
Standard deviation	77	75	70	65	76
Min	139	124	124	121	155
Max	646	562	546	592	540

**Table 10:** Diameter distribution of PVA-CS nanofibers obtained from the solution of PVA-CS (the ratio being 4–3) with an ethanol-acetic acid ratio of 15–45 at a needle-collector distance of 140 mm, a voltage of 28 kV, and a variation of the feed rate from 0.1 to 0.4 mL/h.

Diameter (nm)	Feed Rate (mL/h)			
	0.1	0.2	0.3	0.4
Mean	311	285	300	335
Standard deviation	75	65	79	83
Min	154	121	134	131
Max	513	592	670	650

**Table 11:** Diameter distribution of PVA-CS nanofibers obtained from the solution of PVA-CS (the ratio being 4–3) with an ethanol-acetic acid solvent ratio of 15–45 at a needle-collector distance of 140 mm, a feed rate of 0.3 mL/h, and a variationin voltage from 25 to 30 kV.

Diameter (nm)	Voltage (kV)					
	25	26	27	28	29	30
Mean	348	334	309	285	307	318
Standard deviation	102	99	91	65	96	114
Min	97	139	110	121	131	120
Max	652	734	600	592	644	689

**Table 12:** The lattice parameters of PVA-CS nanofibers obtained from electrospun solutions with different ethanol-acetic acid ratios.

Lattice Parameters		Axial Lengths [Å]			Angles [°]			Cell Volume [Å^3^]	Crystal-Linity (%)
		a	b	c	α	β	γ		
Powder	CS	15.7371	8.3352	3.0609	90	90	90	401.5017	48.29
	PVA	15.2596	5.2416	9.7092	90	97.188	90	770.4844	57.69
Ethanol-acetic acid	0–60	12.6928	3.5873	10.8013	90	95.237	90	489.7613	60.64
	5–55	7.6994	16.7927	6.8328	90	96.878	90	877.0803	54.64
	10–50	15.9762	5.5865	9.3954	90	93.823	90	836.6833	62.36
	15–45	12.7499	7.2561	8.637	90	92.457	90	798.3136	57.45
	20–40	16.0564	4.1401	11.3214	90	93.814	90	750.9244	52.22

**Table 13:** DSC data for the thermal desorption of PVA-CS nanofibers at different ethanol-acetic acid ratios in electrospinning solutions.

Ethanol-Acetic Acid Ratio	ΔH_PVA_ (J/g)	χ_PVA_ (%)	ΔH_CS_ (J/g)	T_g_ (°C)	T_m_ (°C)
0–60	57.22	38.15	5.86	75	222, 247
5–55	42.23	28.15	11.19	75	220, 249
10–50	44.09	29.39	9.79	84	220, 250
15–45	38.72	25.81	13.82	75	222, 251
20–40	53.73	35.82	2.56	87	223, 252

**Table 14:** The stages of thermal decomposition and weight reduction of powder and nanofiber samples.

Degradation Stages		Powder		PVA-CS Nanofibers with Different CH_3_COOH/C_2_H_5_OH Ratios				
		PVA	CS	60/0	55/5	50/10	45/15	40/20
First stage	Range (°C)	25–202	25–177	25–172	25–172	25–172	25–172	25–172
	Peaks (°C)	112	67	52; 114	52; 112	52; 112	52; 107	52; 112
	Weight loss (%)	3.04	5.21	5.42	7.34	5.63	8.58	5.63
Second stage	Range (°C)	202–367	177–462	172–377	172–377	172–377	172–377	172–377
	Peaks (°C)	297	302	267	272	272	267	272
	Weight loss (%)	83.37	77.04	63.79	64.19	62.94	61.40	64.31
Third stage	Range (°C)	367–527		377–547	377–547	377–547	377–547	377–547
	Peaks (°C)	422; 442; 462		429; 459	422; 437	427	422; 437; 457	427
	Weight loss (%)	10.32		17.64	18.43	16.72	16.97	16.97

**Table 15:** Parameters of tensile properties of PVA-CS nanofibers.

Ethanol-Acetic Acid Ratio	Tensile Strength [MPa]		Elongation at Break [%]		Young’s Modulus [MPa]	
	Vertical	Horizontal	Vertical	Horizontal	Vertical	Horizontal
0–60	5.9 ± 1.0	5.7 ± 0.6	14.0 ± 1.7	17.1 ± 0.5	343 ± 34	289 ± 74
5–55	5.6 ± 1.5	1.6 ± 0.4	12.1 ± 1.6	13.3 ± 1.1	425 ± 73	246 ± 35
10–50	4.7 ± 0.4	1.9 ± 0.4	11.8 ± 1.5	14.0 ± 2.1	427 ± 50	270 ± 40
15–45	7.9 ± 1.0	4.9 ± 1.0	13. ± 0.5	16.4 ± 0.8	546 ± 32	333 ± 24
20–40	4.5 ± 0.9	2.9 ± 0.2	12.7 ± 2.0	14.1 ± 1.9	398 ± 48	330 ± 29

## Data Availability

The data presented in this work are available upon request from the corresponding author.
